# Tattoo Pigment in Breast Tissue: A Rare Case of Pigment Deposition in Gynecomastia

**DOI:** 10.7759/cureus.86961

**Published:** 2025-06-29

**Authors:** Garrett M Cail, Taylor F Faust, Michael B Steadman, Tony L Weaver

**Affiliations:** 1 Department of Research, Alabama College of Osteopathic Medicine, Dothan, USA; 2 Plastic and Reconstructive Surgery, Huntsville Hospital, Madison, USA

**Keywords:** dermatology, gynecomastia, lymphatic system, reconstructive surgery, tattoos

## Abstract

Gynecomastia with associated tissue pigmentation is rarely reported in the literature. We report a rare case of a 39-year-old male with gynecomastia and unexpected pigmentation within breast tissue, presumed to be related to extensive tattoos. This unusual presentation of gynecomastia, combined with visible pigment deposits in breast and lymphatic tissue, introduces a novel intersection between seemingly unrelated findings. Gynecomastia, a benign enlargement of male breast tissue, is typically idiopathic or hormonally driven. In this case, surgery revealed tattoo-like pigmentation within fibroadipose tissue and lymph nodes. Histopathology confirmed the pigment to be consistent with tattoo ink, raising the possibility of migration via lymphatic channels. While tattoo ink complications are well documented in dermatology, this is the first known report of pigment deposition in male breast tissue in the context of gynecomastia. This raises questions about tattoo pigment migration and potential implications in tissue remodeling or pathology.

## Introduction

Tattooing involves the introduction of pigments into the dermis, resulting in a permanent design. It is generally considered safe; however, complications may occur due to the pigments themselves or the application process. Pigment-related complications include reactions ranging from minor irritation to more serious conditions requiring medical intervention, such as granulomas, lymphadenopathy, and, rarely, malignancies [1,2]. Specific pigment colors have been linked to various adverse effects in specific cases. Red ink has been associated with hypersensitivity reactions, green with chromium, yellow with cadmium, and blue with cobalt [3].

Gynecomastia is the benign proliferation of glandular tissue, stroma, or fat in the male breast [4]. It affects over 50% of neonates, adolescents, and men aged 50-69 years [4]. Most cases are idiopathic and benign, but hormonal imbalance, systemic illness, and medication use can be contributing factors [5]. While often a source of emotional distress, some patients experience pain and discomfort, and, in rare instances, gynecomastia may be associated with malignancy.

We present a unique case in which pigment deposition was discovered during gynecomastia surgery, with histopathological analysis revealing tattoo ink in both the lymph nodes and surrounding tissue. This raises the question of whether tattoo pigment migration might play a role in the development of gynecomastia in some individuals.

## Case presentation

A 39-year-old male presented with bilateral upper breast fullness consistent with gynecomastia. He had previously undergone sleeve gastrectomy and lost approximately 60 pounds, but the breast enlargement persisted. He denied testicular pain or atrophy, recent illness, illicit drug use, anabolic steroid use, alcohol abuse, and prescription medication use.

Physical examination revealed excess tissue in the upper outer quadrants of the breasts bilaterally, measuring over 4.5 cm in thickness. According to the medical team involved in the patient’s care, hormonal studies, including testosterone, follicle-stimulating hormone, luteinizing hormone, thyroid-stimulating hormone, free T4, and prolactin, were reportedly within normal limits, although specific lab values were not available for inclusion. The patient had multiple tattoos over both upper extremities but had not experienced any pigment-related skin reactions or complications.

Given the lack of identifiable secondary cause and failure of conservative management, he was scheduled for bilateral reduction mammoplasty using direct excision and ultrasound-assisted liposuction (VASER liposuction).

**Figure 1:**
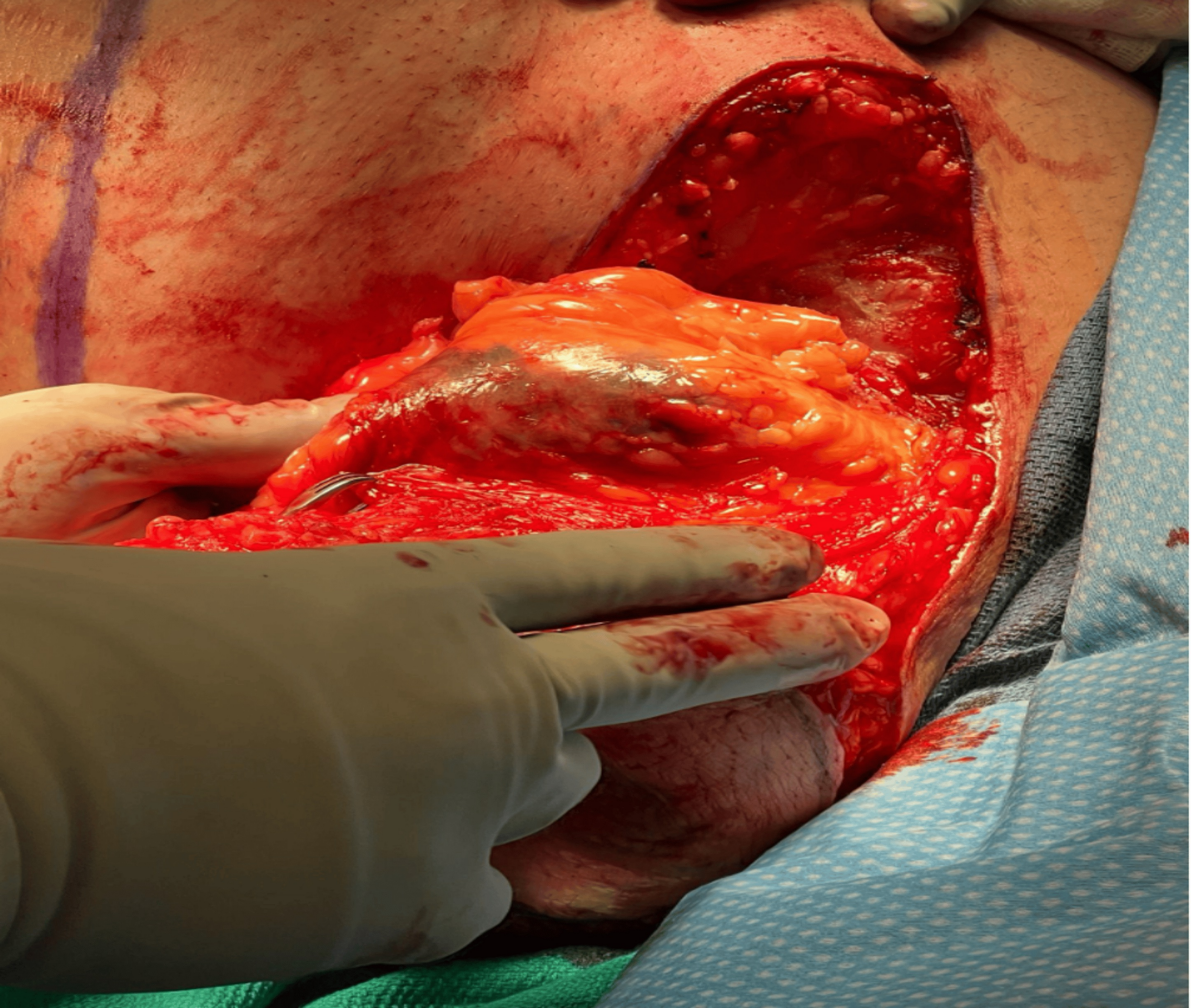
Intraoperative visualization of the lipomatous tissue masses featuring abnormal pigmentation.

Intraoperatively, well-encapsulated lipomatous tissue was identified. Within the excised tissue, prominent lymphatic and vascular structures were noted, and unusual pigmentation was seen, appearing similar in color and pattern to the tattoo ink (Figure 1). This pigmentation was observed intraoperatively in both breasts, although the photographic documentation provided was only of the left side. Tissue samples were submitted for pathological analysis to rule out malignancy and assess for reactive lymphadenopathy.

The left breast specimen weighed 503.6 g and measured 16.5 × 15.0 × 4.3 cm; the right breast weighed 354.2 g and measured 18.5 × 12.0 × 3.5 cm. Both specimens consisted of fibroadipose tissue and skin. Upon sectioning, no discrete masses were identified. However, multiple lymph nodes were present and appeared darkly pigmented.

Histological findings based on the pathology report described multiple lymph nodes and surrounding adipose tissue containing coarse, granular black pigment. No malignancy, granulomatous reaction, or atypical lymphoid proliferation was identified. No histological images were available for inclusion.

## Discussion

Tattoo complications range from minor infections and pruritus to more serious systemic reactions. Infections may include cellulitis, impetigo, tetanus, tuberculosis, hepatitis, Molluscum contagiosum, and atypical mycobacterial infections [6]. Other pigment-associated reactions include hypersensitivity, granulomas, sarcoidosis, photosensitivity, and pseudolymphomas [6]. Granulomas form as macrophages engulf and attempt to isolate foreign pigment, while pseudolymphomas involve polyclonal lymphoid infiltration [6].

Pigment migration to regional lymph nodes is a known occurrence, especially with black ink. This migration can mimic metastatic disease and is most frequently reported in axillary nodes [6]. Though previously described in cases involving female breast tissue or during sentinel lymph node biopsies for breast cancer, the deposition of tattoo pigment within male breast tissue in the setting of gynecomastia has not been documented.

Gynecomastia is typically diagnosed clinically and confirmed with imaging and laboratory evaluation [7]. When pain or emotional distress is significant, treatment may involve medical therapy (e.g., tamoxifen, which has shown a 70% resolution rate [8]) or surgical intervention [9].

While our patient had no identifiable secondary cause of gynecomastia, the pigment deposition discovered intraoperatively and confirmed histologically introduces a novel potential factor. Although a direct causal relationship between tattoo pigment and gynecomastia cannot be established from this single case, the pigment’s presence in the adipose and lymphatic tissue of the breast raises intriguing questions. It is possible that chronic low-grade inflammation or tissue remodeling in response to pigment deposition may play a role in the persistence or development of gynecomastia in select patients.

Additionally, in women, tattoo pigment has been known to mimic microcalcifications on mammograms and can present challenges during radiologic evaluation [10]. The potential for tattoo ink to affect imaging or tissue interpretation in men remains largely unexplored.

## Conclusions

To our knowledge, based on an extensive literature review on gynecomastia and its surgical correction, this is the first reported case describing tattoo pigment deposition within both the breast tissue and lymph nodes of a male patient undergoing surgery for gynecomastia. Pigment migration from heavily tattooed areas likely occurred via lymphatic drainage. Although the clinical significance of this finding remains unclear, the potential relationship between tattoo pigment and breast tissue remodeling warrants further investigation. As tattoos become increasingly common, understanding their systemic and tissue-level effects, especially in locations not typically associated with tattoo ink, will be important. Clinicians should consider this possibility when evaluating unexplained cases of gynecomastia, particularly in patients with extensive tattooing.
